# Supplementary material for: Identification of Novel Tumor Pyroptosis-Related Antigens and Pyroptosis Subtypes for Developing mRNA Vaccines in Pancreatic Adenocarcinoma
Source: Biomedicines. 2024 Mar 25;12(4):726. doi: 10.3390/biomedicines12040726 (PMC11048009; doi:10.3390/biomedicines12040726)
Supplement: Supplementary file 1 [file biomedicines-12-00726-s001.zip › Supplementary figure legend.pdf]

Supplementary figure legend

Figure S1. Study flow chart.

Figure S2. Comparison of differences in enrichment scores of 28 immune cells among three pyroptosis subtypes in the TCGA-PAAD cohort (A) and PACA-CA cohort (B). * $P < 0.05$, ** $P < 0.01$, *** $P < 0.001$.

Figure S3. Comparison of immune scores, stromal scores, and ESTIAMTE scores calculated by ESTIAMTE among three pyroptosis subtypes in the TCGA-PAAD cohort (A) and PACA-CA cohort (B). * $P < 0.05$, ** $P < 0.01$, *** $P < 0.001$.

Figure S4. Functional enrichment analysis of genes in the turquoise module in WGCNA. A. Bar graph shows the top 20 enriched functions and pathways of genes, including the results of enrichment analysis from the GO, KEGG, WP, and Reactome databases. B. Network diagram shows the relationship between various enriched pathways.
