# Supplementary figures and images for: Identification of Novel Tumor Pyroptosis-Related Antigens and Pyroptosis Subtypes for Developing mRNA Vaccines in Pancreatic Adenocarcinoma
Source: Biomedicines. 2024 Mar 25;12(4):726. doi: 10.3390/biomedicines12040726 (PMC11048009; doi:10.3390/biomedicines12040726)

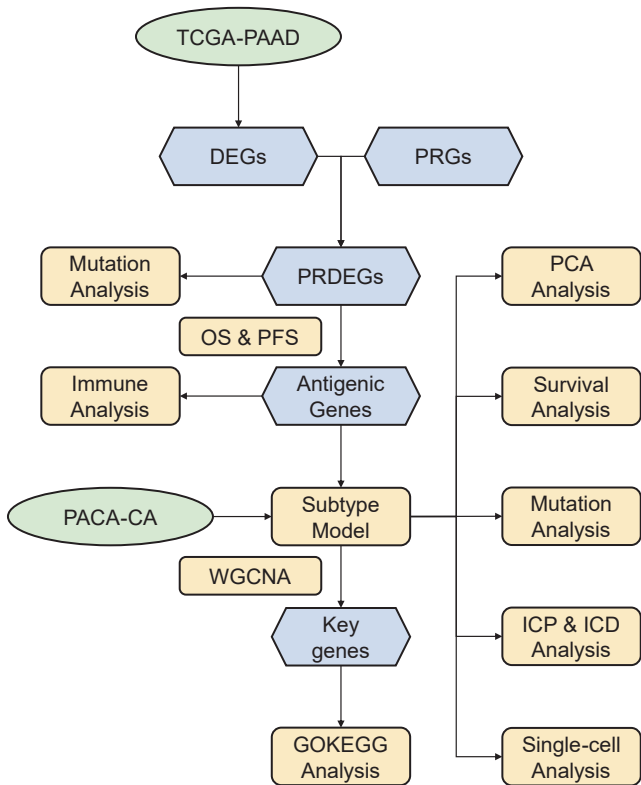

Supplement: Supplementary file 1 [file biomedicines-12-00726-s001.zip › Figure S1.pdf]

A

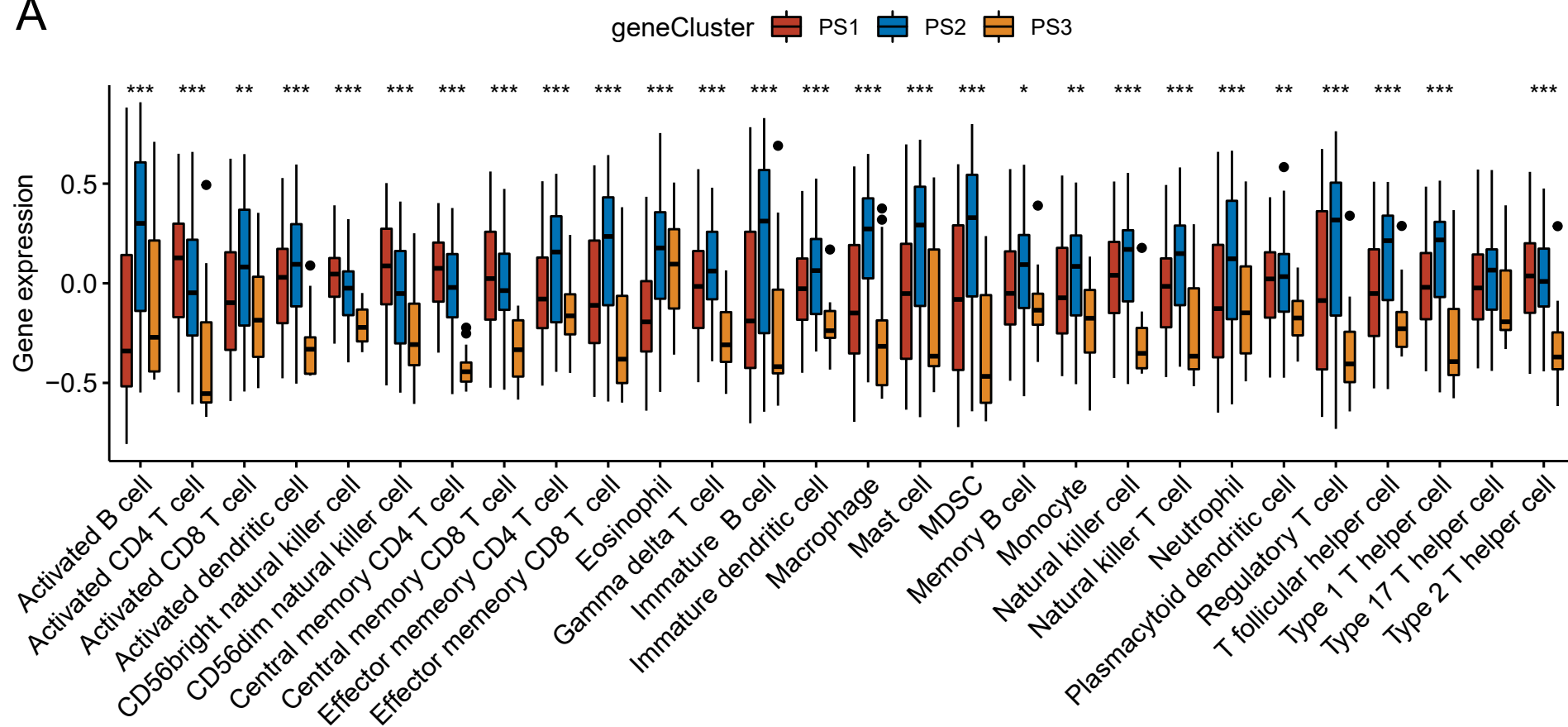

B

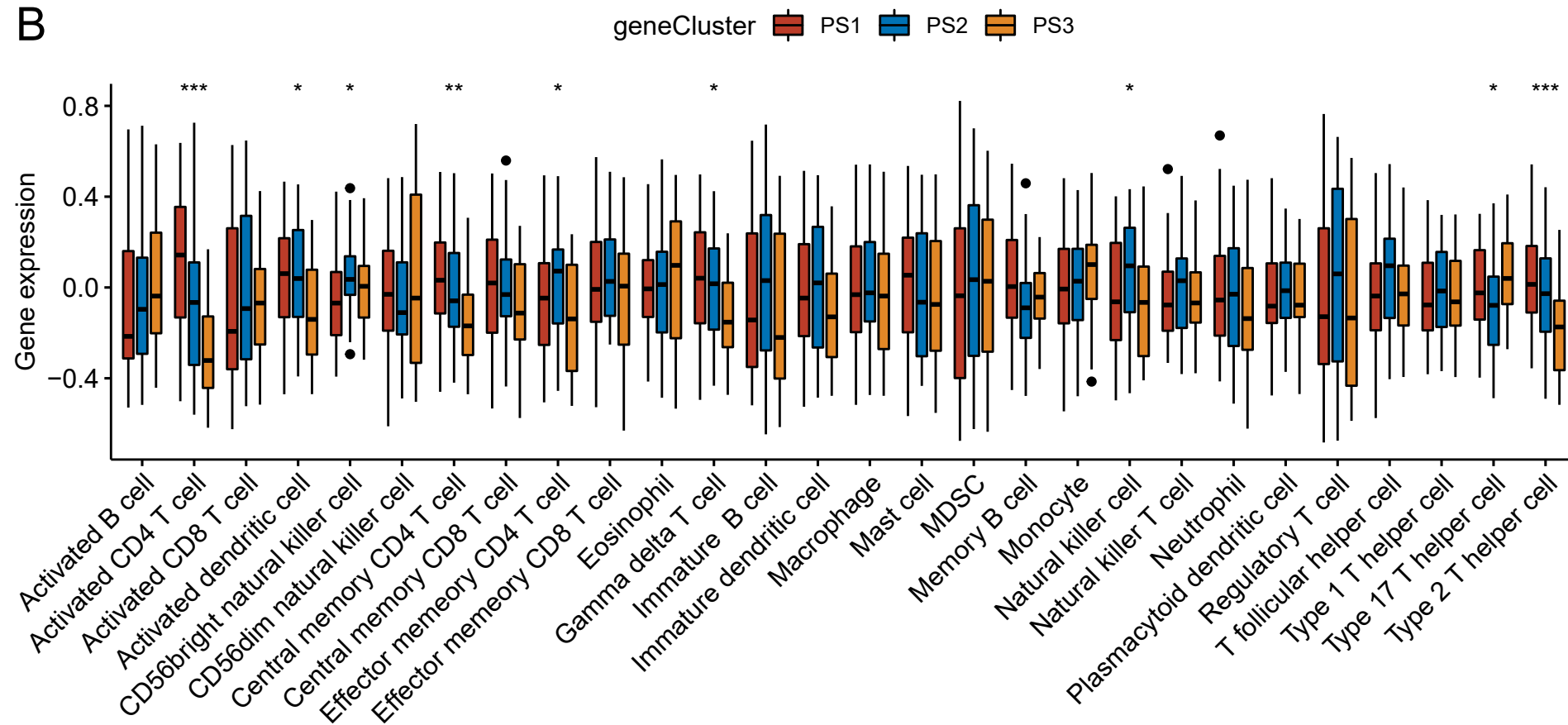

Supplement: Supplementary file 1 [file biomedicines-12-00726-s001.zip › Figure S2.pdf]

A

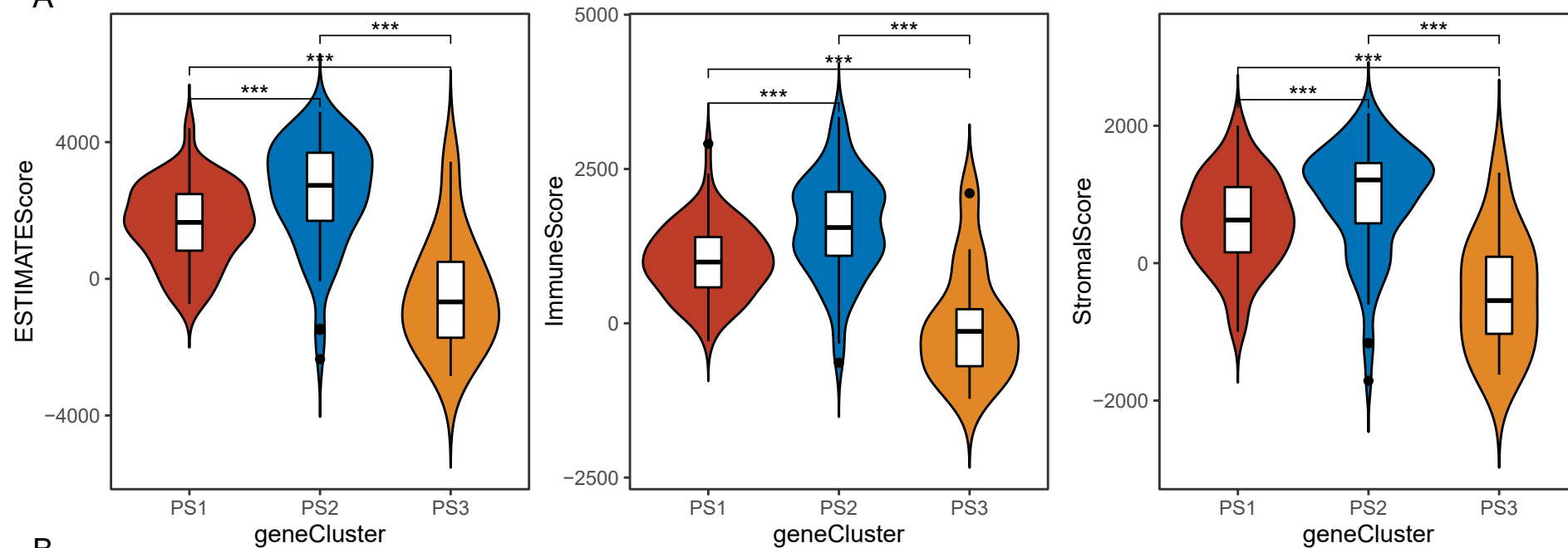

B

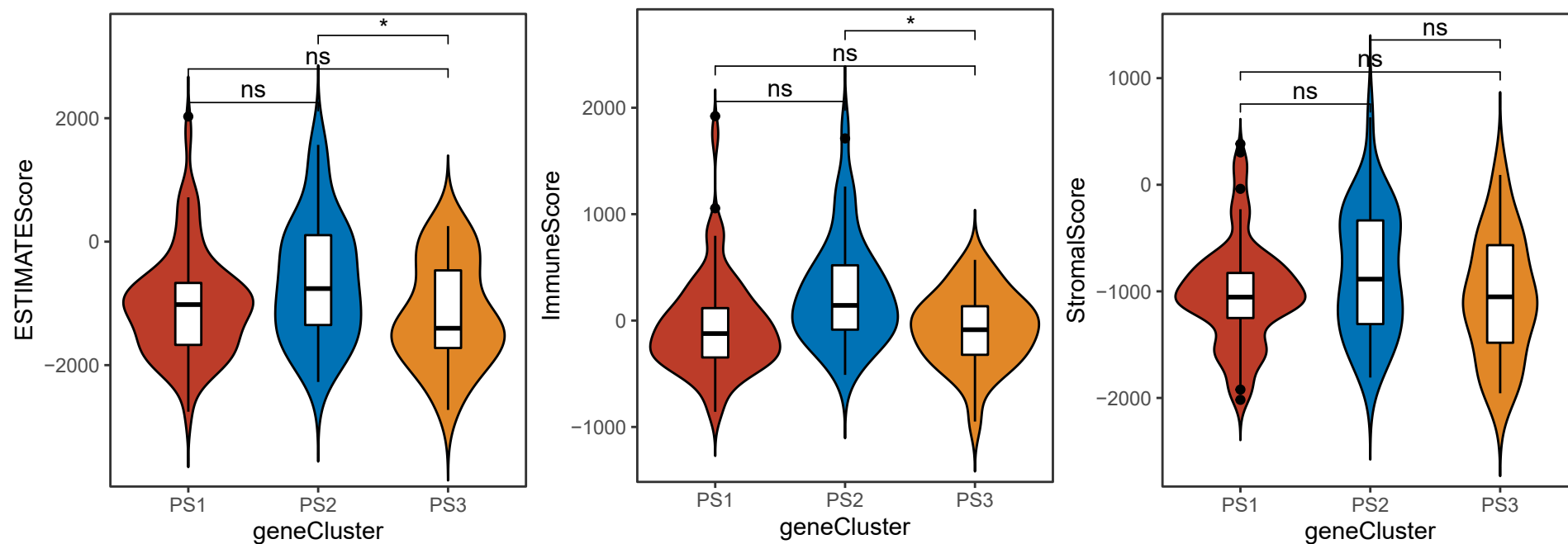

Supplement: Supplementary file 1 [file biomedicines-12-00726-s001.zip › Figure S3.pdf]

A

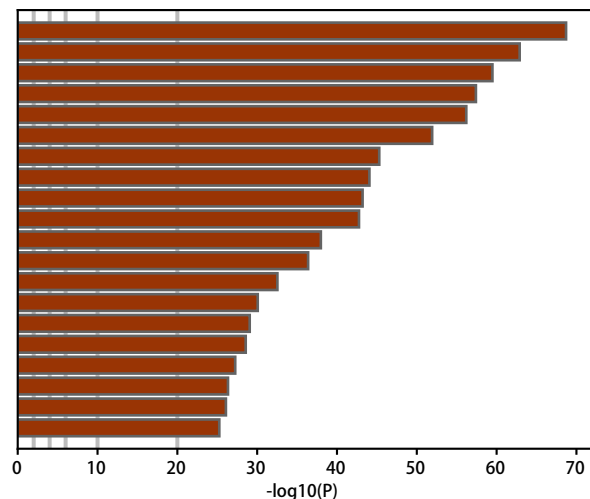

B

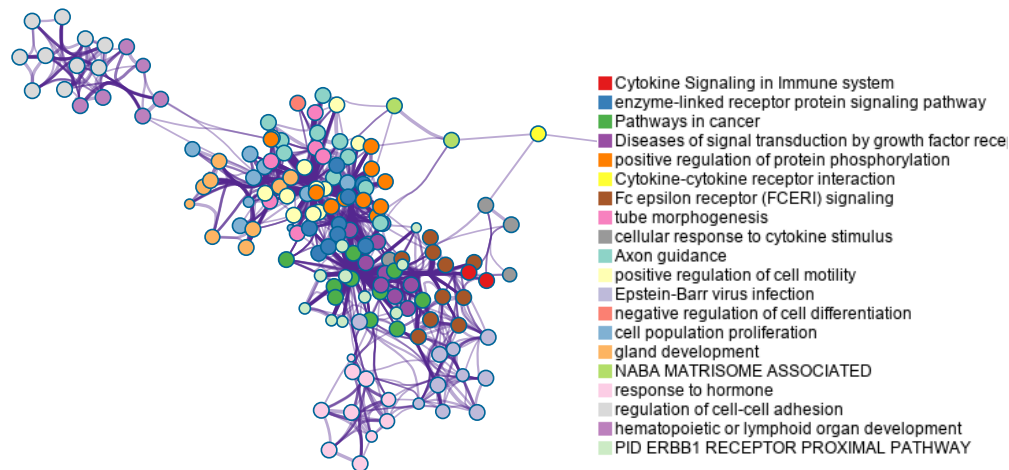

Supplement: Supplementary file 1 [file biomedicines-12-00726-s001.zip › Figure S4.pdf]
